# Cavitation and Other Deformation Instabilities in Plastic Deformation of Semicrystalline Polyethylene Modified with Paraffin Wax

**DOI:** 10.3390/polym17020202

**Published:** 2025-01-15

**Authors:** Alina Vozniak, Zbigniew Bartczak

**Affiliations:** Centre of Molecular and Macromolecular Studies, Polish Academy of Sciences, Sienkiewicza 112, 90-363 Lodz, Poland; alina.vozniak@cbmm.lodz.pl

**Keywords:** semicrystalline polymers, polyethylene, tension, deformation mechanism, deformation instability, cavitation, microbuckling, slip instability, lamella fragmentation

## Abstract

The deformation behavior and instabilities occurring during the drawing of high-density polyethylene (HDPE) were investigated using wide- and small-angle X-ray scattering (WAXS and SAXS) and scanning electron microscopy (SEM) in plain HDPE and paraffin wax- and/or chloroform-modified samples. In contrast to neat HDPE, the modified materials demonstrated strongly suppressed cavitation. However, regardless of cavitation, the tensile deformation of all samples was found to be governed by crystallographic mechanisms active in the crystalline lamellae, supported by shear in the amorphous layers, i.e., the same mechanisms as those operating in other deformation modes. In addition to cavitation, which seems to be a tension-specific phenomenon that does not have a major effect on the deformation sequence, two other important deformation instabilities were observed: microbuckling followed by development of lamellar kinks, at true strain of e = 0.3–0.4, and slip localization instability leading to lamellar fragmentation at e > 0.6. These instabilities were found to be common and very important steps in the deformation sequence, greatly influencing the deformation behavior and occurring in similar strain ranges in both compression and tension, regardless of cavitation. In contrast, cavitation is not able to substitute or significantly modify the main deformation mechanisms, and, furthermore, it does not compete with the main instabilities associated with crystalline lamellae, especially microbuckling; therefore, it may be considered a tension-specific side effect that is not essential for plastic deformation behavior, although it can still significantly affect the final properties and appearance of the drawn material.

## 1. Introduction

The unique chemical and physical properties of semicrystalline polymers make them an important class of materials. Their mechanical properties and related deformation habits are among the most favorable for many applications. Therefore, the deformation behavior of semicrystalline polymers and the associated molecular mechanisms have been extensively studied over the last few decades; see reviews, e.g., [1,2,3,4,5]. The high deformability of semicrystalline polymers is attributed to their long chain structure and unique morphology consisting of periodic stacks of thin lamellar crystals separated by layers of an entangled amorphous phase, which are further organized into higher-level structures, e.g., spherulites. Such multi-level morphology results in very complex deformation and related structural evolution, which ultimately leads to a highly oriented morphology. Three distinct models of semicrystalline polymer deformation have been proposed: the crystallographic slip model [1,6,7,8], the stress-induced partial melting–recrystallization model [9], and the interpenetrating network model [10]. Unfortunately, none of them can adequately describe the complete deformation sequence, and there are still some questions remaining.

The crystalline and amorphous layers are tightly connected by numerous chains crossing the interfacial boundaries (e.g., tie molecules, loose loops, or loose chain ends) and strongly entangled with other chains in the amorphous phase. These chains ensure the load transfer across the lamellar stack and are therefore called stress transmitters (ST) [2]. This strong coupling of adjacent crystalline and amorphous layers forces them to deform jointly despite their very different mechanical characteristics, which generally allows the integrity of the interfaces to be maintained. Recent studies highlighted the very important function of the amorphous phase, including ST chains, as an active element capable of tuning the response of the crystalline component [10,11,12,13,14,15,16,17]. Furthermore, high local stresses and interactions between crystalline and amorphous layers may also induce some instability in the deformation process. The main deformation instabilities found in semicrystalline polymers are associated with the phenomena of cavitation, microbuckling, and lamellae fragmentation (see [18,19] and references therein). They are all related to the amorphous layers and their topology-related characteristics, which determine the stress response as well as interactions with adjacent lamellar crystals [18,20]. Instabilities can prove beneficial to further deformation because they can lead to new deformation routes that may turn out to be more efficient in accommodating strain than those previously available.

Cavitation, i.e., extensive formation of small voids in a material, is commonly observed during drawing or bending of semicrystalline polymers but practically has not been observed in compression or shear. Cavitation, most often accompanied by stress whitening, clearly deteriorates the properties and appearance of products made of semicrystalline polymers, although it may appear beneficial in some specific applications, e.g., membranes. Cavitation was initially considered a marginal phenomenon accompanying deformation. However, due to the growing requirements of the industry, it has been investigated more extensively in recent years, see the reviews, e.g., [5,21,22]. Cavitation usually begins at small strains near the yield point but can extend up to large strains when a fibrillar structure develops [22]. At low strains, nanometer-size voids are created in equatorial regions of spherulites inside the amorphous layers that dilate under tensile force normal to the layering (so-called lamellae separation mechanism). On the other hand, Men et al. [23] postulated that cavitation can also be initiated in the polar region of spherulites by opening the boundaries of the blocks forming crystalline lamellae. It is apparent that there is a competition between the generation of cavities in the amorphous layers and the plastic deformation of the crystalline lamellae: cavitation was postulated to occur when the critical stress for cavitation is smaller than or equal to the yield stress of crystals [21]. This competition is controlled by the lamellae thickness, determining the yield stress, and the topology of the amorphous phase, including the entanglement density and the concentration of stress transmitters [24,25]. Rozanski et al. observed that cavitation is related to the presence of the free volume pores in the amorphous phase and can be suppressed by filling these pores with a low-molecular-weight liquid agent [26,27].

Microbuckling is the second deformation instability observed in the low-strain range. It consists of the undulation of crystalline lamellae together with adjacent amorphous layers, which swiftly develop into bigger folds or angular kinks [18,19,28,29,30,31,32,33,34,35]. These may further evolve towards a chevron-like morphology [30]. Microbuckling in semicrystalline polymers is analogous to the well-known buckling phenomenon observed at very different length scales in a variety of materials characterized by layered morphology [20]. It occurs in materials exhibiting a large difference in stiffness between hard and soft layers, usually in response to the compressive stress acting parallel to the layering. Hence, it is mainly observed in compression. Nevertheless, in some materials that demonstrate high Poisson’s ratio and strong interlayer coupling (such as block copolymers or semicrystalline polymers), the buckling phenomenon was observed to occur also under the action of a tensile force perpendicular to the layers [19,30,31,32,36]. Microbuckling in semicrystalline polymers during their drawing was believed to compete with cavitation that is initiated in amorphous layers of the same orientation, but at slightly lower strains—cavitation was expected to alleviate the compressive stress component along layering, which is the driving force for microbuckling, thereby suppressing the latter [30]. However, recently, both cavitation and microbuckling instabilities have been observed to occur during tensile deformation of high-density polyethylene (HDPE) [19], which demonstrates that cavitation does not actually sufficiently reduce the stresses promoting microbuckling.

Microbuckling instability, although limited to a relatively small fraction of lamellae with a specific orientation, can appear very important for further deformation because the rapid reorientation of lamellae in the kinks facilitates their further deformation by crystallographic slip, a mechanism not available in their original orientation. This leads to a new, easier deformation path. When microbuckling is intense, such as in compression, the microbuckling-activated deformation can show up macroscopically as the second yield point, observed at e = 0.3–0.4 [18,33,37].

Another important deformation instability is the fragmentation of lamellae into smaller crystalline blocks, which usually starts at true strain around e = 0.6 [10,18,33,38,39]. The fragmentation process ultimately leads to the destruction of the original lamellar morphology, which is replaced by a new, preferred crystal ordering along the direction of plastic flow. The mechanism of fragmentation was suggested first by Young et al. [40] who attributed this to the development of a so-called “coarse chain slip”, which eventually can lead to the fragmentation of lamellae into small blocks by chain slip strongly localized in coarse steps. It was discussed further by Galeski et al. [41]. The slip instability leading to its localization and, consequently, to lamella fragmentation, in addition to irregularities in lamella thickness or inhomogeneities in plastic resistance, may be additionally stimulated by other plastic inhomogeneities, e.g., by local stress concentrations induced on the lamella faces by taut ST chains [18,33] or by small voids present in the adjacent amorphous layer due to preceding cavitation [42,43,44]. These stress concentrations apparently lead to an earlier localization of crystallographic slip and extensive lamella fragmentation.

The aim of this work was to investigate the tensile deformation behavior of semicrystalline polymers, including deformation instabilities that can occur during the process when cavitation is impeded. HDPE was chosen as a model material because it is the simplest polymer in terms of chemical structure, has well-known properties, and is widely used in a broad range of applications (e.g., packaging, automotive, distribution pipes, high-strength fibers, medical applications, etc.) due to its well-balanced mechanical properties, chemical resistance, low cost, or recyclability [45]. To achieve the goal, the tensile deformation of plain HDPE, demonstrating extensive cavitation, was compared with that of HDPE modified by blending with paraffin wax and/or swelling with chloroform, in which cavitation is strongly suppressed. Both plain and modified materials have identical microstructure and properties of the crystalline component, while they differ markedly in properties of the amorphous phase, including the concentrations of entanglements and ST chains. This study is an extension of our previous work [19], in which the samples of HDPE swollen with solvents, such as n-hexane or chloroform, were used. That modification resulted in a reduction in the stress during drawing, which effectively hindered cavitation. However, it did not alter the molecular network topology in the amorphous phase or the fraction of ST chains, in contrast to the modification applied in this work. Therefore, in the present study, we expected to gain further insight into the effect of cavitation on other deformation instabilities that may occur during drawing, including cavitation–microbuckling competition, and also to observe the influence of varying concentrations of ST chains on slip instability. Tensile deformation of modified HDPE was additionally compared to that observed during cavitation-free compression, studied earlier [18,33,35,37]. The presented results support our previous findings [19] that the deformation in tension and in compression goes according to the same mechanisms and, moreover, the same crucial instabilities occur in similar strain ranges, except only cavitation that is exclusive for tension. Cavitation, if present, has a rather limited influence on other mechanisms and deformation behavior, although it can accelerate the lamellae–microfibrils morphological transformation.

## 2. Materials and Methods

### 2.1. Material and Sample Preparation

The material used in this study was high-density polyethylene (HDPE) provided by Basell (Rotterdam, The Netherlands), with a molecular weight of M_w_ = 170,000 and M_n_ = 28,900, a melt flow rate (MFR) = 0.2 g/10 min (2.16 kg, 190 °C), and a density of 0.962 g/cm^3^. Paraffin wax with a melting point of 58 °C (Park Sci. Ltd., Northampton, UK) was used for the preparation of the blend with HDPE. Chloroform (>98.5%; T_m_ = −63.5 °C; T_b_ = 61.2 °C; ρ = 1.49 g/mL; POCH, Gliwice, Poland) was used as received for swelling modification of the HDPE and the blend samples.

The blend of HDPE with 6 wt.% of paraffin wax was prepared by melt blending in a co-rotating twin-screw extruder (L/D = 25; Zamak, Skawina, Poland) at T = 190 °C. The 1 mm thick films were prepared from pellets of neat HDPE or the blend with paraffin wax by compression molding at 190 °C and 50 MPa, followed by quenching between cold aluminum blocks.

Dumbbell-shaped specimens for tensile experiments were cut from the compression-molded films, with a thickness of 1 mm, 10 mm width, and a nominal gauge length of 10 mm, in accordance with ISO 8256 standard.

Modification by swelling of solidified samples was carried out by immersing the tensile specimens in a chloroform bath for 72 h at room temperature to achieve full penetration of the amorphous phase by the solvent. The concentration of chloroform in the sample was estimated from its weight increase. It was found that samples of plain HDPE, as well as the HDPE/wax blend, were already completely swollen after 20–24 h of immersion. The chloroform fraction in the modified materials estimated after 72 h of immersion was 7.6 vol.% and 7.3 vol.% for plain HDPE and the blend, respectively.

### 2.2. Mechanical Testing

The mechanical properties of the materials studied were evaluated in uniaxial tension experiments using a tensile testing machine (Model 5582, Instron, Norwood, MA, USA) equipped with the 2 kN loading cell. The specimens, according to the ISO 8256 standard, were used in all experiments. Tensile tests were performed at room temperature at a constant crosshead speed of 0.01 mm/s.

Prior to the drawing, a rectangular grid of dot markers was printed on the specimens. The digital images of the specimens were recorded at fixed time intervals during the tensile test and additionally right after unloading and 48 h later, when the strain recovery was completed, for determination of the change in strain with time and the distribution of the local strain in the sample along the direction of drawing. The local true (Hencky) strain *e_loc_(x)* was calculated from the increase in distance between adjacent markers, measured in photographs, according to the following formula [46]:

(1) $$e_{loc}\left(x\right)=\int_{l_{o}}^{l}\frac{dl(x)}{l(x)}=ln\;(l(x)/l_{o})$$

where *x* is the coordinate along the tensile axis, *l_o_* is the initial distance between a pair of adjacent markers before deformation, and *l*(*x*) is the corresponding distance in the deformed sample. The local true strain was alternatively assessed from the measured reduction in the specimen width [46]:

*e_w_* (*x*) = *ν*^−1^ *ln*(*w*(*x*)/*w_o_*) (2)

where *w_o_* is the initial width, *w* is the local width at a given location *x* in the deformed sample, and *ν* is the Poisson ratio. The distribution of local strain along the direction of drawing at a given sample extension was obtained using *e_loc_*(*x*) values calculated from photographs of the sample taken under load, as well as immediately after unloading and after another 48 h, required for relaxation and strain recovery.

The true strain at the point of maximum elongation *e_H_* (*t*) = *max*(*e_loc_*(*x*,*t*)) and the actual specimen width at the corresponding location were also estimated from the photographs taken at regular time intervals during drawing. The true stress was then calculated by dividing the load recorded by the tensile machine at a given time stamp by the corresponding actual cross-section of the sample according to the following equation:

(3) $$\sigma_{H}\left(t\right)=\frac{F(t)}{A(t)}=\frac{F(t)}{A_{o}}{\left(\frac{w_{o}}{w(t)}\right)}^{2}$$

where *F*(*t*) is the force, *A*(*t*) is the actual cross-section area, and *A_o_* is the initial cross-section area of the sample, respectively. The ideal uniaxial deformation, implying an equal reduction in sample width and thickness, *w_o_*/*w* = *d_o_*/*d*, was assumed in Equation (3). This assumption was positively verified for very similar samples in a wide strain range [19,47]). The true stress–true strain curves were constructed using the calculated values of *e_H_* and *σ_H_*.

### 2.3. Characterization

Small-angle X-ray scattering (SAXS): The lamellar structure of samples was examined via two-dimensional small-angle X-ray scattering (2-D SAXS) using a laboratory line consisting of a low-divergence CuK_α_ radiation microsource (Xenox SA, Grenoble, France) coupled with a multi-layer collimating mirror and a system of 4 crossed scatterless slits (Xenox), a 1.2 m long vacuum camera, and a solid-state area detector (Pilatus 100 K, Dectris, Baden-Daetwill, Switzerland). The well-collimated X-ray beam had a square cross-section of approx. 0.6 × 0.6 mm^2^ in the sample plane. Details are described in Ref. [18].

**Figure 1:**
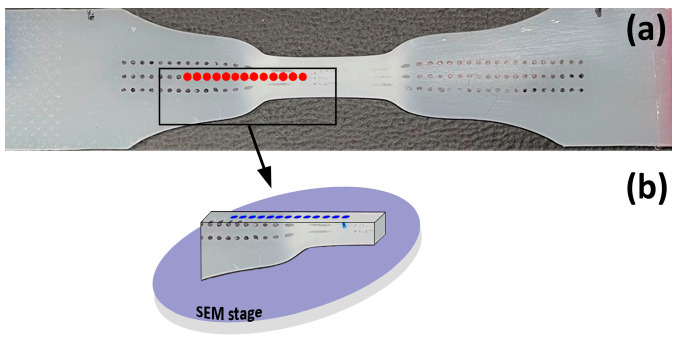
(**a**) The photograph of the HDPE tensile sample drawn to the extension of 25 mm. Black dots are the markers printed on the sample prior to deformation for estimation of the local strain. The red dots represent the points where 2-D SAXS images were collected. (**b**) The schematic view of the cut of the sample for SEM observation—blue dots indicate the points of further SEM observations.

The samples for SAXS experiments were prepared via uniaxial drawing in a tensile testing machine until a neck was formed and the natural draw ratio inside the neck was reached, typically to the extension of about 25 mm. Then, still, under load, the central part of the sample, including the neck, was fixed via a special rigid metal frame holder inserted into the space between the grips of the tensile testing machine in order to maintain the elongation and prevent strain recovery [48]. Next, the specimen with deformation just fixed in the central part by the holder was released from the grips of the tensile testing machine and transferred to the SAXS line, where a series of SAXS images were collected at successive points along the extension axis at increments of about 1 mm (Figure 1a). The next day, the sample was released from the holder and allowed to relax and partially recover the strain for at least another 48 h. The recovered sample was scanned once again with an X-ray beam to obtain 2-D SAXS images at the corresponding points. For each SAXS image, the local strain was determined based on the coordinates of the observation point and the photographs of the sample printed with deformation markers, taken in the loaded and recovered state.

Wide-angle X-ray scattering (WAXS): Analysis of the crystalline structure of the materials was performed in reflection geometry using a diffractometer Aeris (Malvern Panalytical, Almelo, The Netherlands) connected to a sealed-tube source of CuK_α_ radiation, operating at 40 kV and 7.5 mA. The CuK_α_ line was filtered using a thin Ni filter.

Differential Scanning Calorimetry (DSC): Thermal analysis was performed using a DSC calorimeter TA 2920 (TA Instruments, New Castle, DE, USA) calibrated with indium. The heating scans at a constant rate of 10°/min, under nitrogen flow, were employed. The weight crystallinity *X_c_* was calculated from the recorded heat of melting [49]:

(4) $$X_{c}=\frac{∆h_{f}}{∆h_{f100}}\cdot 100\%$$

where Δ*h_f_* is the measured heat of melting of the sample, and Δ*h*_*f*100_ = 293 J/g is the heat of melting of 100% crystalline PE [50]. The volume crystallinity *X_v_* was calculated using the following formula [49]:

(5) $$X_{v}=\frac{X_{c}\rho_{a}}{\rho_{c}-(\frac{X_{c}}{100})(\rho_{c}-\rho_{a})}$$

where *X_c_* is the weight crystallinity (wt.%), while *ρ_c_* = 1.0 g/cm^3^ and *ρ_a_* = 0.85 g/cm^3^ are densities of the crystalline and amorphous phases, respectively.

Scanning Electron Microscopy (SEM): The specimens for SEM observations were prepared by permanganic etching according to the procedure developed by Olley et al. [51,52]. In the first step of preparation, the internal morphology of the sample was exposed by cutting along the plane of interest with a fresh razor blade or a microtome (Tesla, Brno, Czech Rep.). Then, it was etched with a fresh solution of 1 wt.% KMnO_4_ in a 1:1 *v*/*v* mixture of concentrated sulfuric and phosphoric acids, typically for 60 min at room temperature. The etched samples were carefully washed [51], dried, and coated with a fine gold layer (Edward Sputter Coater, Crawley, UK) before being examined with a scanning electron microscope JEOL JSM-6010 LA (JEOL, Tokyo, Japan) operating in high vacuum mode and at an accelerating voltage of 10–20 kV. The samples were probed with SEM at the observation points along the tensile axis, close to the points previously examined with SAXS; cf. Figure 1b. For each observation point, the corresponding local strain was estimated based on the coordinates of the SEM stage and a photograph of the sample printed with deformation markers, upon which estimations were verified by measuring the sample thickness in low-magnification SEM micrographs. Details of the procedure are given in Ref. [19].

## 3. Results and Discussion

**Figure 2:**
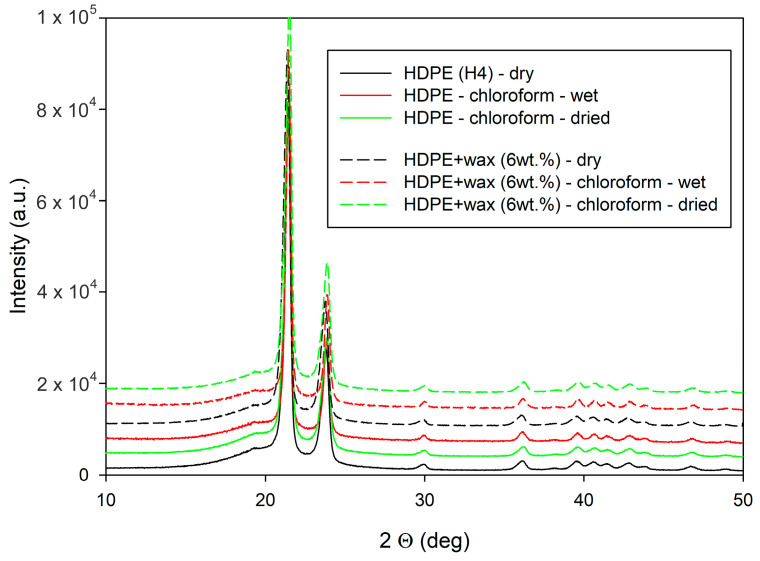
X-ray diffractograms of the HDPE neat and blended with 6 wt.% of paraffin wax: dry and swollen with chloroform.

The initial structure and morphology of the neat and modified HDPE were characterized using WAXS, SAXS, and DSC. X-ray diffractograms presented in Figure 2 show that all samples studied in this work exhibited the same crystal structure—the orthorhombic form—typical for polyethylene. The virtually identical diffractograms of plain and modified materials clearly demonstrate that the crystal structure was not altered by the modification of HDPE, neither with paraffin wax nor by swelling with chloroform. Moreover, the same crystal structure was also observed in the swollen samples after drying to remove the solvent.

As demonstrated by the results obtained from DSC and SAXS measurements, presented in Table 1, the blend of HDPE with 6 wt.% paraffin wax exhibited crystalline lamellae of similar thickness and a slightly lower melting temperature, but the overall crystallinity was reduced, and amorphous layers were thicker compared to plain HDPE. Previous studies [18,44] indicated that when the wax concentration in the blend was low, the paraffin wax did not show any phase separation, and the blend consisted of lamellae of pure polyethylene and interlamellar amorphous layers constituting a homogeneous mixture of polyethylene and wax molecules. The presented results suggest the same structure in the samples studied here. Wax molecules dispersed in the amorphous phase act as a diluent and cause an increase in the thickness of the amorphous layer. This dilution also leads to a decrease in the density of the molecular network and, consequently, to a reduction in the concentration of ST molecules in a solid material, which effect has indeed been experimentally confirmed [18,53]. The reduction in the effective network density is relatively small—from 4.3 × 10^26^ m^−3^ in plain HDPE to 3.9 × 10^26^ m^−3^ in the blend with 6 wt.% of wax, respectively [53].

Similarly to modification by blending with wax, the swelling modification of solid samples with a solvent, such as chloroform, affects exclusively the amorphous phase—the solvent can only penetrate and swell the amorphous layers but does not dissolve or modify the crystalline lamellae [54]. Consequently, the lamellae remain intact, while the amorphous layers increase their thickness, which is confirmed by the DSC and SAXS results presented in Table 1—the melting temperature and therefore the lamella thickness remain unchanged, whereas the long period increases, indicating an increasing thickness of the amorphous layers. This change is reversible, and the structure returns to the initial state upon drying the sample. However, in contrast to melt-blended HDPE, the swelling modification is carried out in a solid material with an already established lamellar structure and topology of the amorphous phase, confined and fixed by adjacent lamellae. Therefore, it cannot modify the existing molecular network and change the effective entanglement density in the amorphous phase. On the other hand, as will be discussed later, the expansion of amorphous layers due to swelling in the solid sample introduces noticeable tensile stresses in the neighboring lamellae, tightly connected to the amorphous layers, which has noticeable consequences on the deformation behavior, especially yielding.

The tensile deformation behavior of neat (dry) HDPE was compared with that of HDPE modified by blending with 6 wt.% of paraffin wax and by swelling of solid samples with chloroform. Unfortunately, during stretching of the swollen samples at room temperature, a small part of chloroform was gradually lost due to evaporation from surfaces—as estimated in the ref. [19], in a relatively long deformation experiment lasting 40 min, the chloroform concentration in the HPDE sample decreased from about 7.6 vol.% to 5.9 vol.%. This change appeared insufficient to restore the deformation habit of the swollen sample back to that characteristic of completely dry material. Therefore, the samples can be considered well swollen, even at the end of the drawing, when the concentration of solvent was lower.

**Figure 3:**
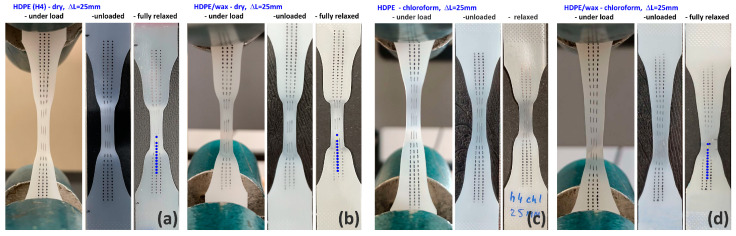
The photographs of the tensile samples deformed to the extension of 25 mm: (**a**) neat HDPE, (**b**) HDPE/wax blend, (**c**) HDPE modified with chloroform, and (**d**) HDPE/wax modified with chloroform. Each sample was photographed under load, right after unloading, and 48 h after unloading (relaxed). Blue dots indicate the points of the samples, which were probed with SAXS. Photographs in (**a**,**c**) were reproduced from the ref. [19]. Copyright 2024, the authors (CC-BY 4.0).

As illustrated by photographs of tensile samples presented in Figure 3, all materials studied in this work formed a neck after passing the yield point, gradually spreading over the entire length of the sample with increasing elongation. The neat HDPE demonstrated strong whitening in the necked zone, while the materials modified either with wax or chloroform did not whiten but instead became transparent in the necked part. Since stress whitening is a common mark of cavitation [21], it can be guessed that cavitation was intense in samples of neat HDPE while probably absent or strongly reduced in those modified with paraffin wax and/or chloroform. Moreover, the neck shoulders in the modified samples, especially those swollen with chloroform, were longer and more diffuse than in the neat HDPE, indicating that the transformation from the initial to the well-oriented structure was more gradual in modified samples than in neat HDPE. The neck shoulders sharpened after unloading and during the relaxation period, which indicates an intensified strain recovery in these transition zones. Rozanski et al. [42,43] postulated that suppressed cavitation in modified samples with low-molecular modifiers like paraffin wax or solvent is related to the filling of free volume pores in the amorphous phase with these molecules, which causes the critical stress of cavitation to increase above the yield stress [42,43]. In addition, as will be shown below, the yield stress in the swollen sample is reduced, which further promotes cavity-free deformation.

**Figure 4:**
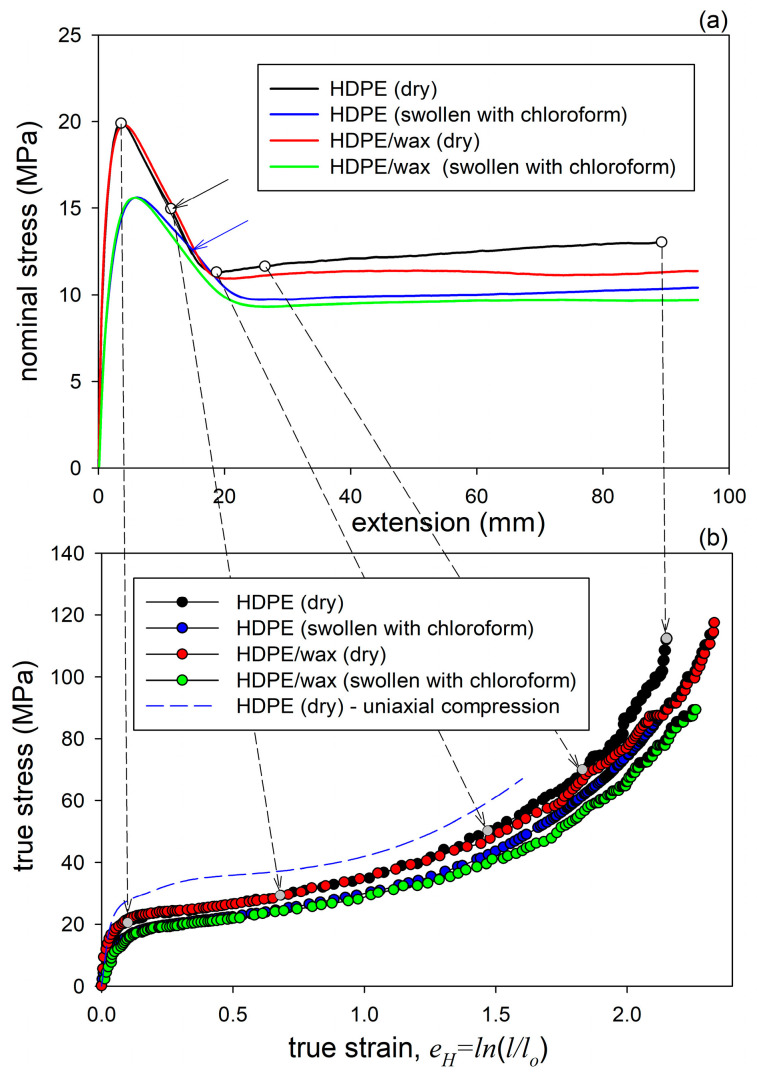
The tensile nominal stress–extension (**a**) and derived true stress–true strain curves (**b**) determined for neat HDPE and HDPE modified with paraffin wax and/or chloroform. True strain was calculated from the change in distance between markers, using Equation (1). The arrows in (**b**) indicate the points corresponding to the characteristic points of the nominal curves marked with open circles in (**a**). In (**b**), the true stress–true strain curve determined in uniaxial compression (data taken from the ref. [35]) is shown with a blue dashed line for comparison.

Figure 4a shows representative nominal stress–elongation curves obtained in the drawing at room temperature. The shape of these curves is typical of uniaxial drawing, with distinct yield tooth followed by a shallow minimum in stress and then the relatively flat part related to neck propagation. Beyond the maximum at the yield point, a small bulge can be discerned in all curves on the right-hand slope of the yield tooth (indicated with arrows in Figure 4a). This feature, often seen in the stress–strain curves of semicrystalline polymers and referred to as the second yield [55,56,57], is commonly associated with a change in the crystallographic slip pattern from homogeneous (fine) to heterogeneous (coarse or blocky slip) that ultimately leads to extensive lamella fragmentation [57]. Both plain HDPE and the HDPE/wax blend show very similar yield stress, and the flow stress in the blend is only slightly lower than in plain HDPE. The similar values of the yield stress are due to the very similar crystalline structure in both plain and wax-modified samples, with nearly the same lamella thickness. The lower flow stress in the blend can be attributed to the smaller network elastic stress due to the lower concentration of chain entanglements in the blend than in plain HDPE. Chloroform-swollen samples of HDPE and the blend demonstrate yield and flow stresses at similar levels in both materials, which is lower than in their dry counterparts before swelling. The yield tooth in the swollen samples is wider, with the maximum shifted towards higher extension compared to dry materials. The lower yield stress of swollen samples was explained by Rozanski et al. [43,44,54,58] by the modification of the stress in the slip plane of crystals—when the amorphous layers dilate along their thickness due to swelling, the ST molecules become stretched, which generates normal tensile stress in the adjacent lamellae [43]. For a given external tensile force, this causes an increase in the resolved shear stress in the slip plane. Consequently, the swollen polymer can reach its yield point at a smaller external force than the dry polymer. Secondly, the expansion of the amorphous layers in the direction parallel to layering in response to swelling is strongly restricted due to the very strong coupling with adjacent lamellae. This generates a lateral tensile stress in the lamella, approximately perpendicular to the slip plane, which, according to the Coulomb yield criterion, applicable to semicrystalline polymers, leads to a reduction in the shear resistance of crystals in the swollen sample [19]. The two above effects together lead to a reduction in the external tensile stress that is required for the yielding of lamellar crystals in the swollen material. As a result, both the macroscopic yield stress and the plastic flow stress are lower in the swollen material than in the dry state.

Because necking leads to an uneven strain distribution along the sample, the interpretation of the nominal stress–extension curves in terms of stress and strain is difficult. Therefore, true stress–true strain curves were constructed based on the force–extension source data and the local strain and cross-section area estimated from photographs taken periodically during drawing, using Equation (1). The obtained true stress–true strain curves are shown in Figure 4b. The curves in Figure 4b were compared with the true stress–true strain curve determined for a dry HDPE sample of the same grade deformed by uniaxial compression [35], shown by the dashed line. It can be seen that tension and compression curves are very similar in shape, which may suggest that the same deformation mechanisms operate in the same order in both tension and compression. Nevertheless, the tensile curves show lower yield stress and plastic flow stress than the compression curve, but this is simply due to the difference in the stress perpendicular to the slip plane. This stress is positive for tension and negative for compression, which, according to the Coulomb yield criterion, causes a decrease or increase in the shear resistance and therefore the yield and flow stresses, respectively [19]. Another important difference is that the compression curve shows a characteristic hump around e_H_ = 0.3–0.4, which is unlikely to be seen in tension. The presence of this bulge in compression has been associated with deformation instability identified as microbuckling of the lamellae, resulting in the development of lamellar kinks [18,33,34,35,37]. The absence of this feature in tensile curves may indicate that microbuckling there is either not as intense as in compression or maybe even absent, being substituted by other micromechanisms. On the other hand, a slight hump was actually observed in the nominal tensile stress—extension curve after passing the yield point, although at a higher strain (e_H_ ≈ 0.6–0.7) than expected for microbuckling. This feature, commonly referred to as the second yield point [55,56,57], may still be related to microbuckling instability, similarly to compression, although delayed, but also to another micromechanism, e.g., lamella fragmentation [57]. Our previous study confirmed that microbuckling was indeed an active mechanism during the drawing of plain HDPE as well as HDPE swelled with solvents, although it was less intense than in compression [19]. The curve of the HDPE/wax blend demonstrates a lower slope than plain HDPE at high strains in the strain hardening range. This slope depends on the response of the amorphous phase, determined by its topology. The lower slope of the curve of the blend confirms then the previous guess that the density of the molecular network in the blend is lower than in plain HDPE.

**Figure 5:**
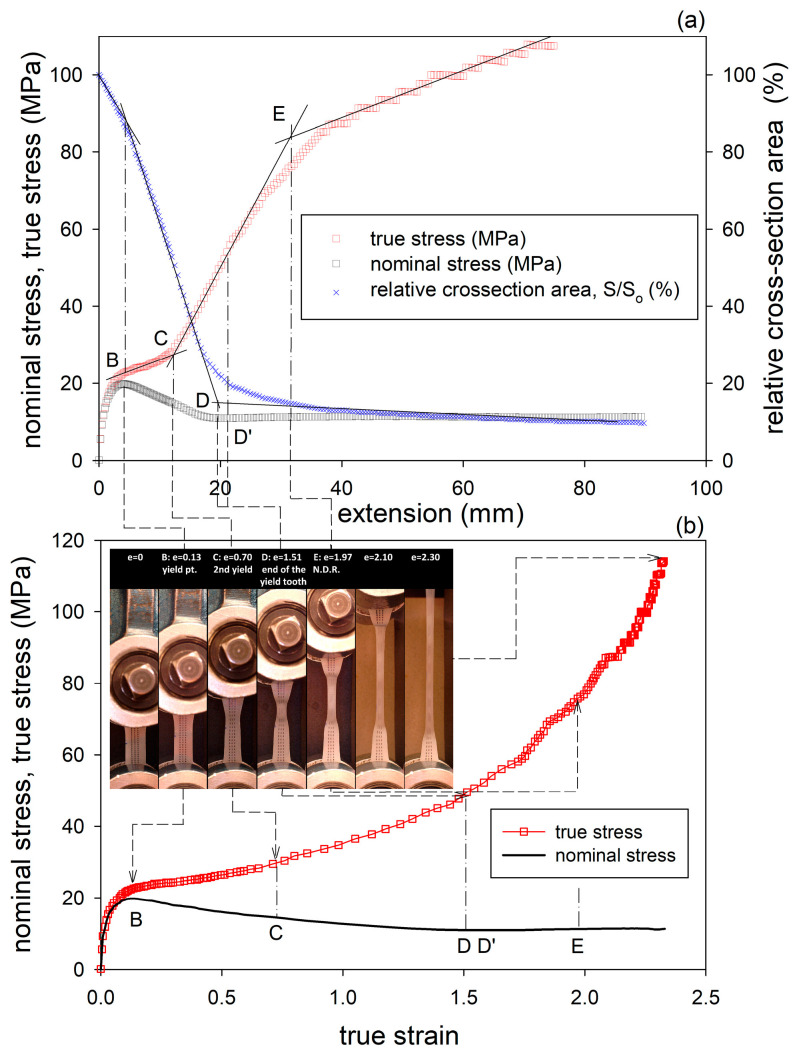
Relationships of stress and relative cross-section area to extension (**a**) and nominal- and true stress to true strain (**b**) determined for a dry HDPE/wax blend. The true strain was calculated from the change in the distance between length markers—Equation (1). The inset in (**b**) shows photographs of the specimen at characteristic strains denoted with the letters B–E.

To gain better insight into mechanical behavior, more relationships were derived from the experimental data in addition to the true stress–true strain curves, including the dependencies of the sample cross-section area or the true stress on extension. An exemplary plot obtained for dry HDPE/wax blend is presented in Figure 5. The dependencies obtained for other samples had a very similar shape. Therefore, they are not presented here (analogous plots constructed for the samples of dry and chloroform-swollen HDPE have already been presented and discussed in Ref. [19]). It can be seen in Figure 5a that the curves of the true stress and cross-section area vs. extension can be easily approximated by several straight line segments whose intersections determine four crossover points, B-E (the first characteristic point A, at e_H_ < 0.05, indicating the end of elastic proportional range [10] is not analyzed here). The macroscopic yield point B, defined by the maximum in nominal stress vs. extension, correlates well with the abrupt decrease in slope in the true stress–extension curve and the first crossover point in the cross-section area vs. extension curve (B’), indicating an accelerated reduction in the cross-section area due to beginning plastic deformation that tends to localize and produce a neck, all occurring in the true strain range e_H_ ≈ 0.1–0.15. The next crossover point, C, seen in the true stress–extension curve at extensions corresponding to the true strain e_H_ ≈ 0.6–0.75, coincides well with the hump on the slope of the yield tooth in the nominal stress–strain curve (the second yield point). From this point on, true stress increases much faster. Such behavior may be associated with some instabilities in the deformation process, such as, e.g., microbuckling [18], the onset of the block slip, or widespread fragmentation of lamellae [10]. These phenomena should result in (at least partially) the release of the constraints that were previously imposed by the surroundings on the deformation of long lamellae and, consequently, in the acceleration of plastic deformation and a faster increase in the true stress due to increasing orientation. At the third crossover point, D (e_H_ ≈ 1.4–1.6), the rapid reduction in the sample cross-section area slows down as a clear macroscopic neck is formed. This corresponds to a shallow minimum in the nominal stress–extension curve, observed at point D’, at a strain slightly exceeding D, as well as to the inflection point observed in the true stress–extension curve. These changes may be a sign of the completion of the process of lamellae fragmentation and the formation of a new crystal arrangement within the neck—the microfibrillar structure. Once created, the neck develops further to a mature form, with only a moderate change in the cross-section but progressively increasing molecular orientation. Thus, the sample reacts with increasing true stress. Eventually, the fourth crossover, E, can be recognized at extensions that correspond to e_H_ ≈ 1.7–2.0, at which the natural draw ratio (NDR) is reached inside the neck. Then, further deformation occurs primarily by spreading the neck along the sample rather than by a significant increase in the deformation of the material within the already necked part. The propagation stage ends above e_H_ ≈ 2, and then further increasing deformation inside the neck is observed, which is accompanied by some rather limited propagation of the neck beyond the narrow-gauge length region. In contrast, the respective true stress–true strain curves (Figure 5b) above the yield point are smooth, do not show any specific features, and only illustrate the strain-hardening behavior, especially at strains exceeding 1.

The positions of the crossover points discussed above are summarized in Table 2. The same points were identified in all samples, although in samples of modified materials, they were observed at strains a little higher than in the plain and dry HDPE. These changes can probably be associated with the altered mechanical response of the amorphous component modified with paraffin wax and/or chloroform, including suppressed cavitation.

In another experiment, the specimens were drawn to an elongation of approximately 25 mm, at which point a mature neck had already developed in the middle of the sample but had not yet reached the full gauge length. In these specimens, the distribution of local true strain along their length was determined under load, right after unloading, and 48 h later, which was the time sufficient for relaxation and strain recovery. The estimations were based on measurements taken from photographs of the deformed specimens imprinted with dot markers. Three distinct regions were identified in each sample: a severely deformed neck, the neck shoulders as the intermediate zones, and much less deformed outer parts away from the neck. It was observed that the neck developed in modified samples at a little higher local strain, and the transition zones (neck shoulders) were longer and more diffuse than in neat HDPE.

**Figure 6:**
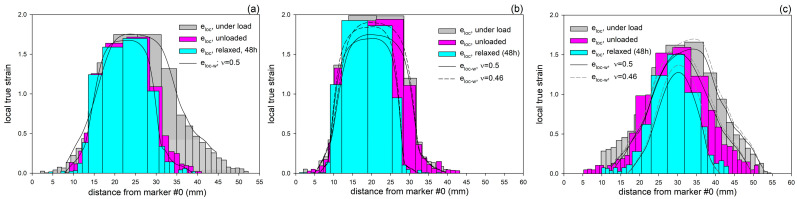
The distributions of the local true strain along the sample length determined in samples of the dry neat HDPE (**a**), dry HDPE/wax blend (**b**), and HDPE/wax blend swollen with chloroform (**c**). The bars illustrate the strain distributions determined from a distance of the length markers printed on the sample, whereas solid and dashed lines show the distribution calculated for the loaded and fully relaxed samples from the local width, assuming the Poisson ratio of ν = 0.5 (solid line) and 0.46 (dashed line). The plot of Figure 6a was drawn after ref. [19]. Copyright 2024, the authors (CC-BY 4.0).

Figure 6 shows the distributions of local strain determined for dry HDPE, dry HDPE/wax blend, and the blend swollen with chloroform (for the results of chloroform-swollen HDPE, see Ref. [19]). In this Figure, the local strains were calculated either from the change in distance between the markers imprinted on the sample (Equation (1))—results presented by bar plots—or from the reduction in specimen width (Equation (2))—shown as the solid and dashed lines, for the Poisson ratio assumed at *ν* = 0.5 and *ν* = 0.46, respectively. It can be seen that for cavitating dry HDPE, the length- and width-based results of local strain agree well when the Poisson ratio *ν* = 0.5 was assumed in Equation (2). However, for non-cavitating materials that were modified with wax or chloroform, a better matching was reached if a lower Poisson ratio of *ν* = 0.46 (the value often reported for HDPE [59]) was assumed. This effect may be attributed to the strong cavitation in the neat HDPE, which brings an increase in the volume of the sample during drawing and counteracts Poisson’s effect.

As already mentioned, in all samples, the greatest deformation was found in the central, necked region, with the local strain almost constant along the length of the neck. In the neck shoulders, the local strain decreased rapidly, while in the outer parts, it was already low and decreased further with increasing distance from the neck. Significant strain recovery was observed, consisting of a relatively little elastic recovery immediately after unloading and a notably stronger recovery process during a 48 h relaxation period. This delayed recovery was primarily related to the elasticity of the molecular network of entangled chains in amorphous layers [16]. During the relaxation and recovery period, the less deformed outer parts of the sample recovered almost completely, whereas the recovery inside the neck was much lower, and the material remained strongly deformed.

**Figure 7:**
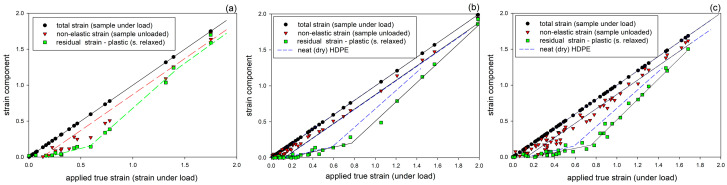
The components of the total strain, determined from the local strain distribution in samples of the neat HDPE (**a**), dry HDPE/wax blend (**b**), and chloroform-swollen HDPE/wax blend (**c**) under load, after load release, and after a 48 h strain recovery period. The lines do not represent any actual relationship and are drawn only to guide the eye. Dashed blue lines, representing the behavior of the neat HDPE, are drawn in (**b**,**c**) for reference. The plot of Figure 7a was drawn after ref. [19]. Copyright 2024, the authors (CC-BY 4.0).

The length-based local strain estimates were replotted against the applied local strain, and the resulting relationships are presented in Figure 7. The plots show three strains: total strain (estimated at a given point when the sample was still under load), non-elastic strain (seen immediately after unloading), and residual strain (remaining after the recovery period; assumed to be permanent). These strain components are additive in terms of the true strain [10,13,16]. The distance between total and non-elastic strains shows the pure elastic (instantaneous, recovering immediately upon unloading) component of strain resulting from the joint response of the crystalline and amorphous phase. It remains constant, e_el_ ≈ 0.12–0.14, in the entire range of applied strain. The gap between non-elastic and permanent strain lines illustrates the component of delayed elasticity associated with the elastic deformation of the molecular network of the amorphous phase. The residual strain, remaining after the recovery period, is a measure of the permanent deformation, mainly related to the irreversible plastic deformation of the crystalline skeleton [13,16]. It appears when the applied strain becomes larger than the elastic strain, defining the yield point, increases with increasing applied strain, up to e_H_ ≈ 0.6 (neat HDPE) or 0.7 (modified samples), and continues to increase even stronger at higher strains. This point of change in plastic response coincides very well with the crossover point C, seen in the stress–strain curves (cf. Figure 5 and Table 2), and is most likely related to the onset of slip localization and subsequent fragmentation of lamellae [10], which leads to a significant reduction or almost complete release of the constraints previously imposed on the deformation of long lamellae and, consequently, easier deformation of short fragments of lamellae or small crystalline blocks resulting from lamellae fragmentation. This turning point shifts towards higher strains in the wax- and/or chloroform-modified systems, suggesting that the fragmentation of lamellae in the modified samples is delayed compared to plain and dry HDPE, perhaps in relation to the suppressed cavitation in these materials. The cavitation, if it occurs, introduces strong stress concentrations at the lamella interfaces, which may promote slip localization and consequently favor lamellae fragmentation [19]. Another change in the recovery behavior is observed at large strains, which is clearly seen in the sample of plain HDPE at e_H_ > 1.0, where the residual strain tends to increase further at the expense of the delayed elastic response, which decreases and eventually stabilizes at a relatively low level due to chain disentanglements occurring at large strains in the already highly oriented amorphous phase, which reduces the network density and hence its elasticity [10,16,60].

The mechanical behavior discussed above is analogous to that of HDPE modified by swelling with hexane and chloroform, reported in our previous study [19]. It was concluded then that such behavior very strongly supports the hypothesis of Strobl and co-workers [10,61] saying that the deformation of semicrystalline polymer and accompanying structural changes are controlled solely by strain. This finding has great practical implications that the structural changes associated with tensile deformation, non-uniform due to necking, do not need to be investigated as a function of sample elongation using a stepwise deformed sample or a large set of specimens stretched to various extensions but can instead be studied using the much simpler approach of scanning the local structure of a single specimen at successive points that have reached different local strain due to the necking process. The structural information gathered in this way should be equivalent to that obtained by examining the structure in a series of samples that have reached different extension [19].

**Figure 8:**
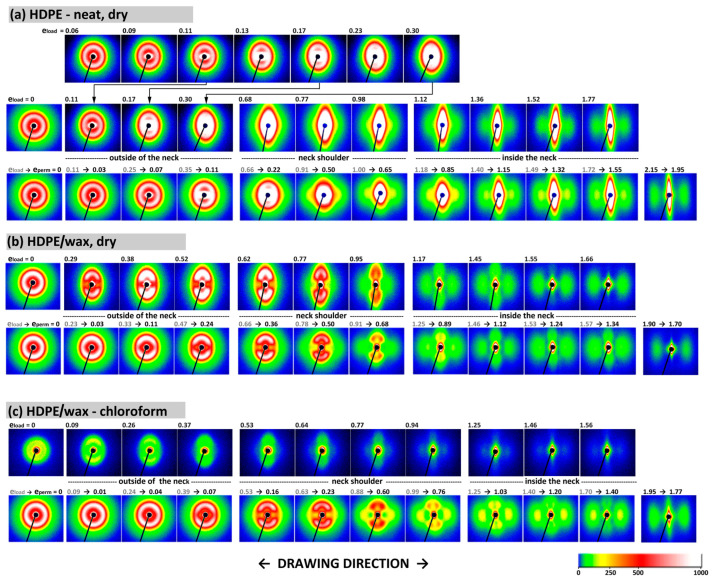
Two-dimensional SAXS patterns of samples of the dry, neat HDPE (**a**), dry HDPE/wax blend (**b**), and HDPE/wax blend swollen with chloroform (**c**), stretched to an elongation of 25 mm. Patterns were recorded in deformed samples still under load—upper rows; after unloading and 48 h of strain recovery—lower rows. For each pattern, the respective local true strain—under load (e_load_) and after recovery period (e_perm_)—are indicated. The last column shows patterns obtained for samples deformed to a high elongation, close to the fracture limit. The drawing direction is horizontal. The patterns of Figure 8a were reproduced from ref. [19]. Copyright 2024, the authors (CC-BY 4.0).

Using the approach outlined above, the lamellar structure of the deformed samples was investigated by two-dimensional SAXS, scanning the specimen deformed to the extension approx. 25 mm along its tensile axis (cf. Figure 1a). The 2-D SAXS patterns obtained for samples of neat HDPE, HDPE/wax blend, and the blend swollen with chloroform under load, and for the same points in unloaded and recovered at least 48 h samples, are shown in Figure 8. The patterns obtained for chloroform-swollen HDPE were very similar to those of chloroform-swollen HDPE/wax blend shown in Figure 8c and are not presented. They were already introduced in Ref. [19].

The deformed neat (dry) HDPE shows under load (top row in Figure 8a) a scattering typical for semicrystalline polymers experiencing cavitation during stretching [5,21], containing a component due to crystalline structure, which is, however, frequently obscured by the second component—scattering caused by nano-voids. The first signature of voids appears along the drawing direction at low strains, near the yield point, and clearly intensifies with strain due to the growing number of cavities. At large strains, a very intense void-related streak is observed perpendicularly to the DR. As already noticed in Ref. [19], the SAXS images of the unloaded sample (bottom row in Figure 8a) evidence that quite a large fraction of cavities healed during the strain recovery period, especially in the range of low and moderate strains. This observation supports the notion that the cavities were located inside the amorphous interlamellar layers [21], partially recovering the strain and shrinking back upon unloading due to entropic forces, which leads to the closure of small cavities. Because the strong scattering by voids obscures heavily the scattering signature of lamellae, it is difficult to observe the evolution of the lamellar structure. Nevertheless, it can be guessed that the original lamellae initially tend to align at some preferred angle with respect to the DR, which led to a faint four-point-like feature that can be discerned at e_H(load)_ ≈ 0.30, unfortunately much concealed behind the strong scattering generated by cavities. A similar weak four-point feature can also be identified in the pattern of the recovered sample at the point of higher applied strain, e_H(load)_ = 0.66, which later recovered to e_H(perm)_ = 0.22. This effect is related to the progressive crystallographic slip causing the lamellae to rotate towards the DR and adopt a preferred orientation, and also likely to microbuckling instability that can occur in lamellae initially oriented perpendicular to the DR, similar to what was observed in compression [18,33,34]. When the strain exceeds e_H(load)_ ≈ 0.6–0.7 (at locations at the beginning of the neck shoulder), a new long-period signature starts to appear along the drawing direction and then intensifies with increasing strain, leading to a final two-line pattern, which can be easily observed at the highest strain (in the necked part of the sample). Such a two-line signal is usually attributed to stacks of short and sheared lamellae or crystal blocks that became preferentially oriented with the chain direction roughly parallel to the drawing direction [62,63], which is the result of instability in crystallographic slip, leading to its localization and, consequently, to severe fragmentation of the original long lamellae. This precursor structure gradually evolves into the final microfibrillar structure, seen at e_H(load)_ ≥ 1.4, due to further action of slip mechanisms. The strain e_H(load)_ ≈ 0.6–0.7, at which the transformation related to slip instability is detected in the 2D-SAXS image, coincides well with the second crossover point, C, discussed earlier, and the hump on the yield tooth, commonly called the second yield point. It also agrees with point C of the Strobl scheme of strain-controlled deformation [10], attributed to extensive lamellae fragmentation.

The 2D-SAXS images of the deformed HDPE/wax blend and the same blend swollen with chloroform look similar to each other. The main difference is that the scattering intensity in the chloroform-swollen HDPE/wax blend, when observed under load, is smaller than in the dry blend as well as in the corresponding images of the relaxed blend sample, which has dried during the recovery period. This effect is caused by the increased density of the amorphous phase after swelling with chloroform (ρ = 1.49 g/cm^3^). Then, the difference in density between crystalline and amorphous phases is smaller, which results in a lower amplitude contrast and, therefore, a lower scattering intensity of the swollen sample. Nevertheless, the recorded images were clear enough to see their most important features.

In contrast to the dry sample of neat HDPE discussed above, no void-related scattering was observed in any of the 2D-SAXS images of deformed samples of HDPE modified with paraffin wax and with chloroform (Figure 8b,c, respectively), neither in the loaded nor in the relaxed state. This, together with the previous observation that modified samples do not show stress whitening, proves the absence of cavitation during the drawing of these materials. The modified samples, especially the blend swollen with chloroform, became instead transparent within the neck. The necked regions remained transparent even after relaxation and drying, regardless of whether the solvent had evaporated from specimens that were gripped to maintain a constant length or from those that had free ends. The suppression of cavitation in modified samples was explained by the effect of filling of free volume pores in the amorphous phase with relatively small molecules of wax or solvent, which increases the critical stress of cavitation above the yield stress [42,43]. In addition, the chloroform-modified samples deform under a lower load than neat HDPE (cf. Figure 4), which implies reduced stresses developed in the amorphous phase, possibly below the critical value required for cavitation [21,59], which additionally favors cavity-free deformation.

Looking at the scattering produced by the lamellar structure, one can easily see that the initial uniform scattering ring related to the non-oriented lamellae (e_H(load)_ = 0) is soon replaced by a four-point feature, starting to appear at relatively small strains, e_H(load)_ ≈ 0.3, when two arcs emerge, centered perpendicular to the DR. Each of the arcs contains two weak maxima away from the DR, which indicates the development of a characteristic four-point pattern. This four-point feature becomes easier to recognize and stronger as the strain increases up to e_H(load)_ ≈ 0.7 and then gradually weakens with further increasing strain. Finally, it almost disappears from the images of loaded sample at e_H(load)_ > 1.4, although it can still be discerned after sample recovery, even for higher applied strain (e.g., in dry HDPE/wax at e_H(perm)_ = 1.34 corresponding to e_H(load)_ = 1.57, or in HDPE/wax-chloroform at e_H(perm)_ = 1.40 corresponding to e_H(load)_ = 1.70). The highly reduced intensity of scattering along the DR and the emergence of a four-point signature at e_H(load)_ ≥ 0.3 indicates that the lamellae, which were originally oriented perpendicularly to the DR, vanished from the structure quite suddenly in this strain range, presumably due to their rapid reorientation to a new position indicated by the highs of the developing four-point pattern. On the other hand, the lamellae that were initially parallel to the DR or aligned diagonally did not change their orientation so rapidly, still contributing to the scattering observed along the arcs viewed perpendicular to the DR. As the four-point pattern evolved, its maxima developed higher and rotated away from the DR with increasing strain, illustrating further lamellae rotation leading to an increasing tilt relative to the DR. No evidence of lamellae fragmentation was found up to e_H(load)_ ≈ 0.7. However, above this strain, the four-point pattern gradually weakened, and a new scattering signature began to appear as an initially weak two-strip signal related to the new arrangement along the DR. As the strain increased, the intensity of this two-line component gradually increased at the expense of a four-point pattern, which, however, did not dissolve completely and could still be discerned in the images even in the range of the highest strains. The appearance of such a two-line pattern is associated with the widespread fragmentation of the original lamellae and rotation of the resulting blocks (short lamella fragments) with the chain direction towards the DR [41]. Eventually, in the range of the highest strain applied, above e_H(load)_ ≈ 1.4 (e_H(perm)_ ≈ 1.1–1.2), the intensity of scattering related to the new arrangement along the direction of drawing became stronger than the intensity of the four-point pattern associated with the preserved remnants of the original lamellar structure, which survived in not very fragmented form, but only significantly reoriented, with the layer planes now almost parallel to the DR.

**Figure 9:**
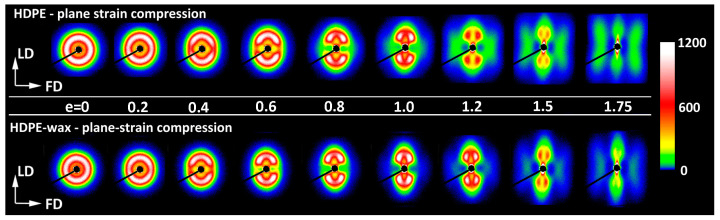
Two-dimensional SAXS images of samples of dry HDPE and HDPE/wax blend (same grades and compositions as in this study), deformed in plane strain compression at room temperature. The true strain applied is indicated for each pattern. The direction of compression (LD) is vertical, while plastic flow (FD) is horizontal. Patterns reproduced with permission from ref. [18]. Copyright 2019, Elsevier.

It was discussed in reference [19] that the evolution of the lamellar structure during tensile deformation of non-cavitating chloroform-swollen HDPE is very similar to the alterations in lamellar structure observed in neat HDPE deformed by plane-strain compression [18]. The same analogy can also be found when comparing the results presented in this study—the SAXS patterns of non-cavitating HDPE/wax blend, either dry or swollen with chloroform, presented in Figure 8b,c—with the patterns of neat HDPE and HDPE/wax blend deformed in cavity-free plane-strain compression deformation mode [18], shown in Figure 9. Cavitation in plane-strain compression is strongly limited by the large compressive stress components, even in the neat HDPE that is prone to cavitation. Comparing the SAXS images of the recovered samples, one can see very similar patterns that developed in both deformation modes in the same strain ranges: a four-point pattern appearing at e_H(load)_ ≈ 0.3–0.4 and persisting up to very large strains e_H(load)_ > 1.7, and a second two-strip scattering component related to a new arrangement, resulting from extensive fragmentation of lamellae and emerging at e_H(load)_ ≈ 0.7–0.8. The shape of the feature associated with the new lamellar order developed in plane-strain compression—the butterfly-like pattern, visible in plain HDPE—differs slightly from the simple two-strip shape observed in deformed tensile samples and indicates a slight tilting of the lamellar blocks in the stacks [63], an orientation that probably resulted from some reverse rotation of blocks during the recovery period. Nevertheless, it can be concluded from this comparison that the same structural transformations—swift reorientation of some lamellae and extensive fragmentation leading to a new ordering—occur in both deformation modes at very similar strains. It is already well documented that a structure leading to a four-point SAXS pattern develops in HDPE during compression as a result of the microbuckling instability, quickly followed by the development of lamellar kinks, which occur at strains around e_H_ ≈ 0.3–0.4 [18,33,34,35]. This instability in deformation causes a rapid change in the orientation of lamellae involved in kinks, especially those initially oriented perpendicular to the direction of the plastic flow but is not accompanied by their severe damage or fragmentation. As a consequence, a noticeable part of the original lamellae is preserved up to high strains. Their significant microbuckling-driven reorientation facilitates further deformation by relatively easy crystallographic slip, activated in a now larger number of lamellae (i.e., those already diagonally oriented plus those that have just been reoriented in the kinks). This manifests macroscopically as a second yield seen in compression as a distinct bulge in the true stress–true strain curve (cf. Figure 4). The great similarity of the 2D-SAXS patterns observed in samples of drawn HDPE modified to prevent cavitation and in compressed materials, where cavitation also does not occur, suggests that the same deformation mechanisms, including extensive crystallographic slip and accompanying deformation instabilities, such as microbuckling and lamellae fragmentation, must have been active and, moreover, they occurred in both deformation modes in the same sequence and at very similar strains. On the other hand, any bulge or hump like that seen in compression around e_H(load)_ ≈ 0.3–0.4 and associated with microbuckling was, in fact, not observed in tensile true stress–true strain curves. This may imply that the microbuckling instability in tension, although suggested by the SAXS results, was probably less intense than in compression and therefore did not manifest itself macroscopically.

**Figure 10:**
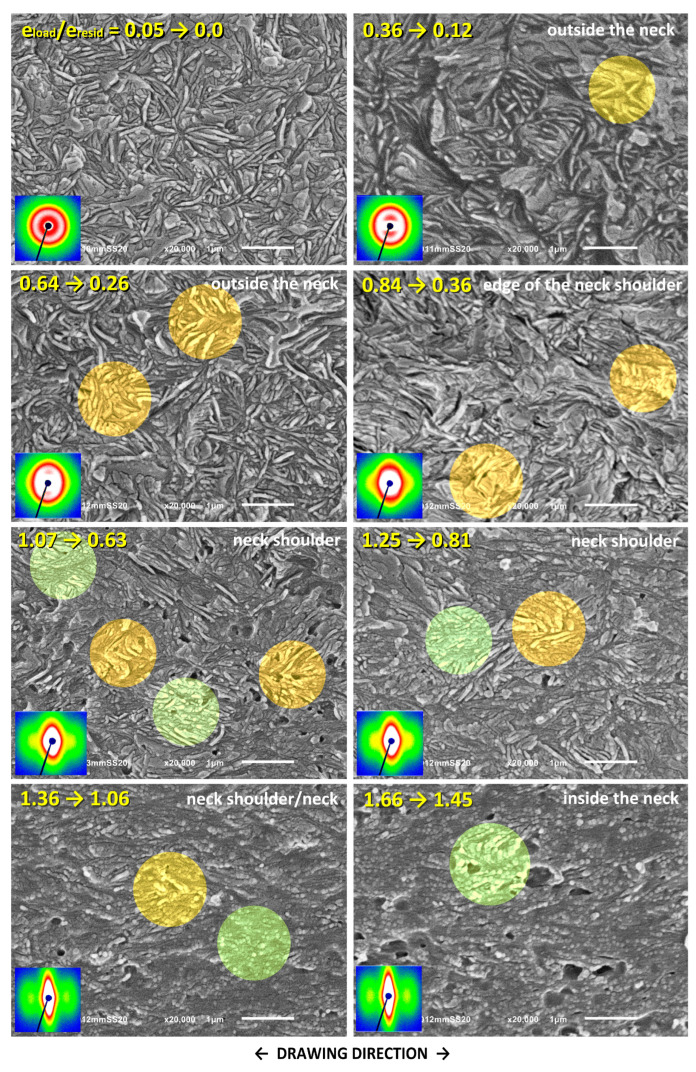
SEM micrographs of the dry neat HDPE drawn at room temperature. Micrographs were taken at different positions along the tensile axis, exhibiting different local strains. The direction of the drawing is vertical. For each micrograph, the estimated true strain under load (e_H(load)_) and the residual true strain remaining after relaxation and strain recovery (e_H(perm)_) are indicated. The insets in the lower left corners show the corresponding 2-D SAXS patterns. Orange circles highlight the lamellar kinks and green—the areas with lamellae fragmentation.

**Figure 11:**
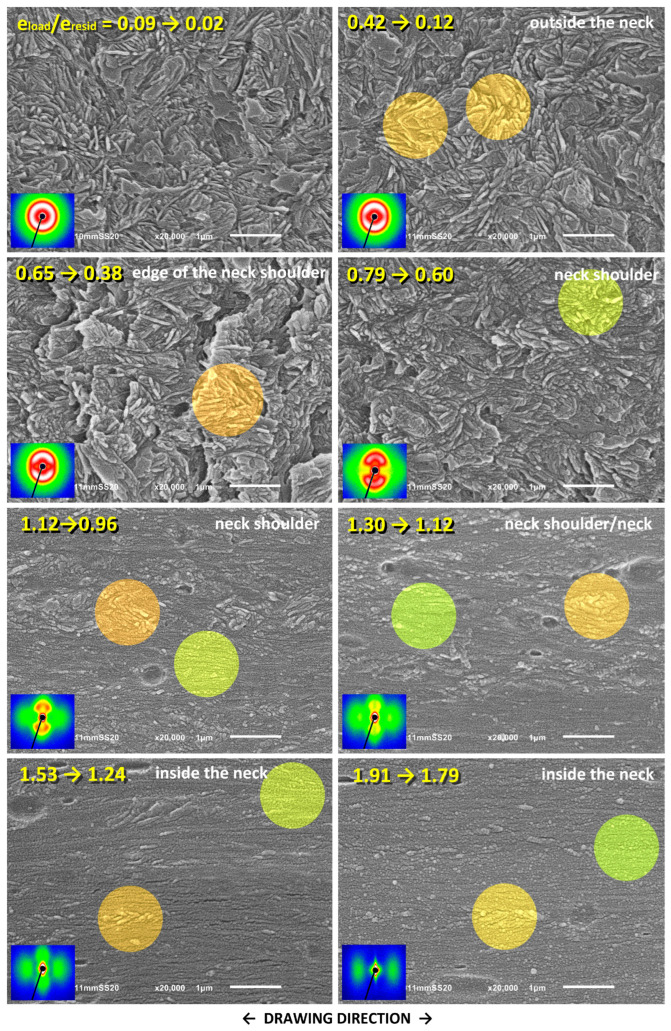
SEM micrographs of the HDPE/wax blend drawn uniaxially at room temperature. See the caption of Figure 10 for details.

**Figure 12:**
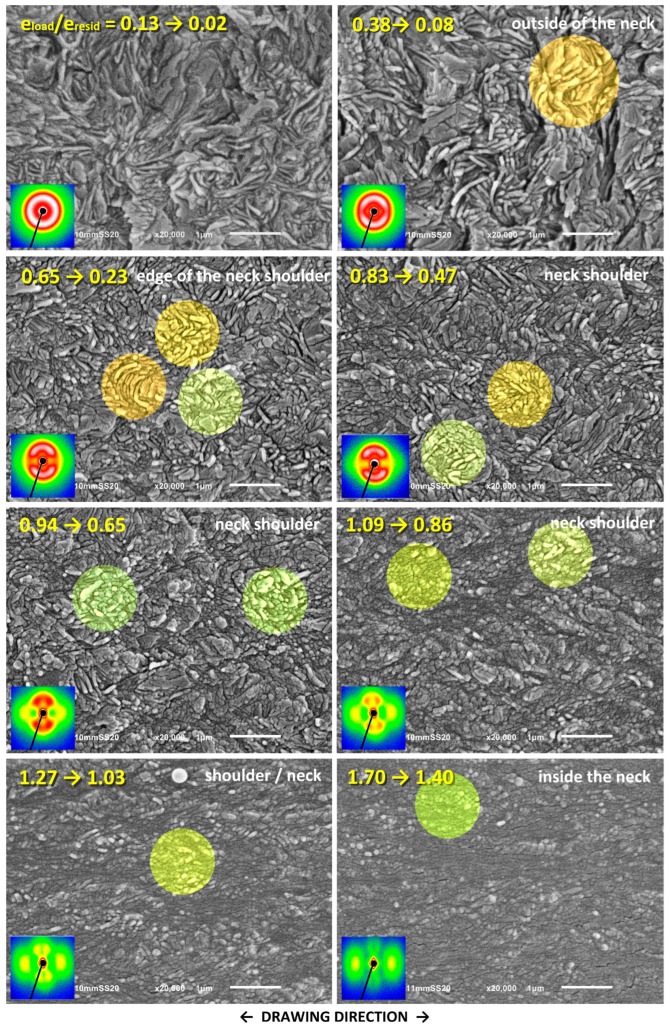
SEM micrographs of the HDPE/wax blend swollen with chloroform, drawn uniaxially at room temperature. See the caption of Figure 10 for details.

The changes in the lamellar morphology accompanying the drawing were examined using a scanning electron microscope (SEM). The same samples, stretched by approx. 25 mm that were previously used in SAXS experiments were taken for microscopic observations. Selected SEM micrographs are shown in Figure 10, Figure 11 and Figure 12. These figures represent a series of micrographs taken at different positions along the tensile axis, close to the points probed earlier with SAXS. The samples were observed after unloading and complete recovery. Therefore, two strains are reported for each observation point: the local strain reached under load, e_H(load)_, and the local residual (permanent) strain remaining after recovery, e_H(load)_, which are given in the corresponding micrographs.

Figure 10 shows the SEM micrographs of the neat HDPE. The changes in the morphology of this material during tensile deformation have been discussed in detail in Ref. [19] and will therefore only be briefly described here. At small strains, e_H(load)_ < 0.2, no specific deformation-related changes in the lamella morphology are observed. At e_H(load)_ = 0.4–0.9, sharp angular kinks can be distinguished, which were formed jointly by several adjacent lamellae (see areas marked in orange in the micrographs). The number of kinks visible in each micrograph is limited because they only arise from lamellae initially oriented perpendicular to the DR. However, the number of lamellae involved in a kink increased with increasing strain. These kinks are similar to the previously observed lamellar kinks that develop during the compression of HDPE due to microbuckling. Therefore, it can be assumed that they are also the result of the same microbuckling deformation instability [18,30,33,34]. This begins as an elastic instability [64], leading to small undulations of the layers that quickly transform into much larger folds or angular kinks. The formation of kinks is accompanied by plastic deformation and possibly very limited damage, particularly at the hinges of sharp kinks [4,33,64,65,66]. The apex angle in the just-formed kink decreases, and its limbs gradually tilt towards the DR due to the intense chain slip activated in the lamellae that have just rapidly changed orientation in the kink [35]. The microbuckling instability seems to start in tension at true strain below e_H(load)_ ≈ 0.4, similar to compression, e_H(load)_ ≈ 0.3–0.4 [18,33,34].

At e_H(load)_ ≥ 0.9, the effect of another deformation instability can be observed. This is instability in crystallographic slip (which is already well advanced, especially in the lamellae oriented diagonally), leading to its localization and consequently to damage of long lamellae and their fragmentation into short fragments (see the areas highlighted in green in the micrographs for e_H(load)_ ≥ 1.07). The extent of fragmentation increases markedly with increasing strain, which finally leads to total devastation of the original lamellar structure and its replacement with a microfibrillar morphology at high strains, well above e_H(load)_ = 1.3. The strain at which the fragmentation is clearly visible in the micrographs coincides with the mark of the abrupt change in the arrangement of the lamella, seen in SAXS patterns above e_H(load)_ = 0.7 (Figure 8a), as well as with the second crossover point, C, identified in the true stress—extension curve, at e_H(load)_ = 0.66 and with the hump in the nominal stress—extension curve (Figure 5 and Table 2). The conclusion is that the slip instability, which further leads to its localization and then to fragmentation of lamellae followed by rotations of the resulting blocks, appears around e_H(load)_ ≈ 0.6–0.7 and then intensifies markedly with increasing strain. This is consistent with the onset of lamellae fragmentation at e_H(load)_ = 0.6, as postulated by Hiss et al. [10] and with our previous findings from compression studies indicating that slip localization and lamella fragmentation occur at e_H(load)_ ≈ 0.6–1.0 [18,33].

There is strong cavitation in neat HDPE during its drawing. The cavities detected with SAXS are generally too small to be observed with SEM. However, they can grow and/or coalesce with each other with increasing strain, reaching sizes large enough to scatter visible light or to be observed by SEM. The voids, most probably related to cavitation, are, in fact, observed in SEM images of deformed HDPE at e_H(load)_ ≥ 0.84 (Figure 10), and the number and size of voids increase with strain, as expected. Although the shape and size of these relatively large voids were most probably altered by chemical etching applied prior to observation, they must be related to actual, pre-existing cavities that were created during the drawing process. As can be seen in Figure 11 and Figure 12, no such features were observed with SEM in samples modified to prevent cavitation.

Cavitation is expected to make slip localization and lamella fragmentation easier and thus intensify it since the presence of cavities introduces large and numerous stress concentrations at the amorphous–crystal interfaces, facilitating slip localization. According to classical calculations by Goodier [67], the stress around a spherical void increases with a factor of at least 2.

Figure 11 and Figure 12 show the SEM micrographs taken for the drawn HDPE/wax blend and the blend additionally modified by swelling with chloroform, respectively. As can be seen in these micrographs, there is no trace of cavitation in either material. Instead, one can distinguish the morphological features that are very similar to those seen previously in the neat HDPE, such as lamellar kinks starting to form at e_H(load)_ ≈ 0.4 (see the orange-highlighted areas in the micrographs) and extensive lamellae fragmentation occurring at e_H(load)_ > 0.7 (see the green-highlighted areas). Similar deformation behavior and related morphological changes were reported in our previous work for non-cavitating chloroform-swollen HDPE [19]. Microbuckling followed by kinking seems to occur slightly more frequently and to be more advanced in all modified materials compared to plain HDPE, while lamella fragmentation, although initially less intense, intensifies markedly at higher strains. However, a fraction of relatively long lamellae that apparently survived the fragmentation, only a little damaged, can still be observed in the modified samples even at large deformation, which is further confirmed by the four-point signature in the corresponding 2D-SAXS images. The morphology, including coexisting relatively long lamellae and small crystalline blocks, is observed up to e_H(load)_ ≈ 1.7, whereas, in plain HDPE, the long lamellae were destroyed much earlier. Another observation is that these small blocks visible at large strains are usually arranged along the linear outlines of former long lamellae, both in neat and modified materials, which is particularly clearly visible in micrographs of the HDPE/wax blend. This indicates that such blocks are the debris of heavily fragmented lamellae due to slip instability and not the result of the strain-induced melting–recrystallization transformation, which was considered in the past to be one of the main mechanisms of tensile deformation [9,68].

The results of SAXS and SEM observations, in addition to confirming the crystallographic slip as the principal deformation mechanism, revealed three noteworthy deformation instabilities occurring during tensile deformation: (1) cavitation, which begins near the yield point (e_H(load)_ ≈ 0.1) and is strong in plain HDPE while practically absent in modified samples; (2) microbuckling instability leading to formation of lamellar kinks and causing a rapid reorientation of involved lamellae (e_H(load)_ ≈ 0.3–0.4); and (3) extensive fragmentation of lamellae due to localization of the crystallographic slip (e_H(load)_ > 0.6, more intense at e_H(load)_ > 1.0), which, at high strains, brings the complete destruction of the original lamellar structure. Mechanical data, supported by SAXS and SEM results, indicate that lamella fragmentation in modified materials, in which cavitation is suppressed, is slightly delayed compared to neat HDPE that experiences heavy cavitation—the second crossover (pt. C) in the true stress–extension curve, indicating onset of the fragmentation, is observed at e_H(load)_ = 0. 0.69–0.75 and e_H(load)_ = 0.66, respectively (see Table 2).

In a previous study [19], an earlier hypothesis regarding a possible competition between cavitation and microbuckling instability was tested, assuming that microbuckling could only be activated when cavitation was suppressed [30]. These instabilities occur in the lamellar stacks initially oriented perpendicular to the DR and at similar strains (e_H_ ≈ 0.1 and 0.3–0.4, respectively). These stacks, when subjected to a tensile force along DR, i.e., perpendicular to the layers, initially respond to this force by dilating the soft amorphous layers, the phenomenon known as lamella separation. In order to maintain a constant volume (the Poisson ratio of the rubber-like amorphous phase is close to *ν* = ½), these amorphous layers must contract laterally. This, however, is strongly limited because of their large lateral dimensions and the very strong linkage with adjacent crystalline lamellae, which can only deform slightly under such stress conditions (elastic response). As a result, compressive stress develops along the layers. The crystalline-amorphous stack may then respond to further drawing either with cavitation within the dilating amorphous layers due to tensile stress perpendicular to layering or with microbuckling instability (undulation of layers, soon developing into a kink) under compressive stress parallel to layers. The actual response depends on the material properties and test conditions (temperature, stress field, and deformation rate). These factors can either impede or promote cavitation in the amorphous phase. Cavitation was expected to relieve this compressive stress component along the layering, thus significantly reduce the driving force for microbuckling so that this instability would only happen in the absence of cavitation [30]. However, the results presented in [19] contradicted this hypothesis since microbuckling instability was, in fact, observed independently of cavitation, both in plain HDPE exhibiting strong cavitation and in samples modified by swelling in which cavitation was limited or disabled. The same conclusions follow from the result presented here—microbuckling was observed in all tested samples, regardless of cavitation. All the above observations indicate that compressive stresses parallel to layering, developed during stretching in suitably oriented stacks, lead to microbuckling and, furthermore, these stresses are not fully alleviated by the formation of voids in cavitating materials and may still be high enough to trigger microbuckling instability. On the other hand, microscopic observations indicate that the kinking of lamellae initiated by microbuckling is not as intense during the drawing when it is accompanied by cavitation as during cavity-free deformation (e.g., in the drawing of suitably modified materials or in compression tests). This supports the notion that cavitation can indeed locally reduce the normal stress, but such a reduction is often insufficient to prevent subsequent microbuckling. Therefore, microbuckling instability involving the undulation of layers followed by the development of lamellar kinks can be regarded as a typical step in the deformation pathway of semicrystalline polymers. This mechanism was found active in both compression [18,29,33,34,35] and tension [28,30,31,32], regardless of cavitation [19].

Microbuckling is limited only to specifically oriented lamellar stacks with layers roughly perpendicular to the DR and can therefore accommodate only a minor strain. Nevertheless, it appears very important for further deformation of the entire material since it results in rapid rotation of the involved lamellae from the initial orientation (where it was extremely difficult to initiate any conventional mechanism, and the deformation was practically locked) to the diagonal orientation that enables deformation by crystallographic slip, which is relatively easy and effective in accommodation of strain. This greatly facilitates further deformation by easy crystallographic mechanisms active from now in almost the entire volume of the material (including the lamellae that were already oriented diagonally and those that have just been reoriented in kinks) [18,33,34]. This can open a new, effective deformation pathway, previously inaccessible.

In contrast, cavitation, which can also occur in the stacks of the same orientation, although able to relieve excessive stress, does not affect the unfavorable orientation of the neighboring lamellae and therefore cannot unlock their plastic deformation, which for sure impedes the overall process.

The last of the deformation instabilities observed in this study leads to the fragmentation of lamellae into small crystalline blocks, which was frequently reported for semicrystalline polymers [10,18,33,38,39,41,42]. That fragmentation is due to instability in crystallographic slip and its subsequent localization. It starts at e_H(load)_ > 0.6 and intensifies markedly with increasing strain, especially at e_H(load)_ > 1.0. The mechanism of fragmentation was suggested first by Young et al. [40], who attributed this to the development of a so-called “coarse chain slip”, which eventually leads to the fragmentation of lamellae into small blocks by chain slip strongly localized in coarse steps. Galeski et al. [41] further explained that the deformation generally proceeds initially by fine (homogeneous) chain slip activated already at the yield point and resulting in gradual reorientation of lamellae accompanied by their substantial thinning. As a result, the lamellae progressively rotate and stretch out towards the direction of plastic flow (equivalent to the DR in tension), undergo lattice rotation tending to align the chain axis also along the DR [40] and become substantially thinned (say, to about 1/3 of initial thickness at e_H_ ≈ 1). Such very thinned lamellae with strongly tilted chains can become unstable—like a stretched thin layer of fluid—and may respond by breaking up and periodic fragmenting, stimulated in both cases by the instability of the interface energy to perturbations. The onset of this instability was postulated to depend on the plastic shear resistances of the layers, the interface stretching resistance, and the layers’ thickness (and, therefore, the long period) [41]. When the lamella (or the fluid layer) becomes very thin, the interface stretching resistance and plastic stretching resistance become comparable, and perturbations decreasing the overall interface energy are likely to grow [41]. These perturbations may arise from uneven thickness or some inhomogeneities in plastic resistance due to structural defects and can lead to local disturbances in the slip process in lamellae, resulting in its strong localization in narrow zones, accompanied by further rapid local thinning of lamella in these regions and, consequently, in fragmentation of long lamellae into small crystalline blocks.

Moreover, the slip instability ultimately resulting in lamella fragmentation can be additionally stimulated by other plastic inhomogeneities, such as, e.g., local stress concentrations induced on the lamella faces by taut ST chains (tie-molecules or mutually entangled loops anchored at opposing interfaces in crystals) [18,33] or by small cavities present in the adjacent amorphous layer [42]. In fact, it was found in compression-deformed polyethylene [18,33] that the critical strain associated with lamellae fragmentation depended on the surface fraction of ST chains at the amorphous–crystal interface—if this fraction was small, fewer but stronger stress concentrations were expected. This apparently caused earlier localization of crystallographic slip, ultimately leading to lamella fragmentation. Rozanski et al. [42,43,44] demonstrated that the presence of the cavities, which can be a source of very strong stress concentrations in adjacent lamellae, also enhances their fragmentation. The same effect was actually observed in [19] and in this study—the fragmentation started at a lower strain and was more extensive in cavitating neat HDPE than in non-cavitating wax- and/or chloroform-modified HDPE (cf. Table 2).

As discussed earlier, the modification of HDPE by melt-blending with 6wt.% of paraffin wax results in a slight reduction in the density of the molecular network and surface concentration of stress transmitter chains crossing the crystal–amorphous interface, while the structure and morphology of the crystalline phase are not changed [53]. This leads to fewer but stronger stress concentrations generated on lamella faces when under load. In contrast, the modification by swelling of plain HDPE or the blend with chloroform was carried out in a solid material with an already established lamellar structure and topology of the amorphous phase, confined and fixed by adjacent lamellae. Therefore, such modification could not change the existing molecular network and the effective entanglement density in the amorphous phase. Consequently, plain and swollen HDPE are expected to show similar molecular networks and concentration of active ST, higher than in the HDPE/wax blend, either dry or swollen.

According to the previous studies [18,33], modification of HDPE with wax reducing the network density should lead to fewer but stronger stress concentrations generated on the lamella faces under load; therefore, slip instability can be expected to occur earlier in the blend than in the case of plain HDPE, provided cavitation is absent. This was, in fact, observed in compression [18,33] and here during the tension of swollen samples: the swollen blend of reduced ST concentration showed the onset of slip instability at e_H(load)_ = 0.69, i.e., earlier than swollen HDPE, e_H(load)_ = 0.75 (Table 2). There is no significant difference between the dry and swollen blend samples (e = 0.70 and 0.69, respectively), which exhibit similar, reduced network density. However, the situation changed when cavitation was active—slip instability and lamella fragmentation started at a noticeably lower strain, e_H(load)_ = 0.66, than in any other sample that exhibited the same or reduced density of the molecular network (swollen HDPE and the blend, dry or swollen, respectively), which demonstrates that the effect of cavitation on lamella fragmentation, introducing strong and numerous stress concentrations, is greater than the influence of reduced ST concentration, resulting in fewer and probably weaker stress concentrations than those arising from presence of cavities. On the other hand, lamella fragmentation, although initially postponed in non-cavitating materials, clearly increases at higher strains. This may indicate that lamella fragmentation, once initiated, proceeds more easily at higher strains if the amorphous phase shows lower network elasticity.

Modification of the samples by swelling leads to a reduction in the stress at yield and plastic flow in both plain HDPE and the blend. This does not seem to affect the stability of lamellae: the non-cavitating dry and swollen samples of the HDPE/wax blend show practically the same critical strain for slip instability.

## 4. Conclusions

The presented results confirmed that the modification of HDPE with a low molecular modifier, either by melt mixing (in the case of paraffin wax) or by its sorption by a solid sample (chloroform), results in a significant reduction in cavitation during tensile deformation, which is otherwise very strong in neat, unmodified HDPE. The most probable mechanism is the filling of the free volume pores in the amorphous phase with modifier molecules, which apparently increases the critical stress for cavitation above the yield stress of polyethylene [26,27]. As in the previous study [19], in addition to conventional mechanisms such as crystallographic slip and interlamellar shear, operating continuously, three significant deformation instabilities were observed in well-defined strain ranges, which were apparently a response to momentary inhibitions in the deformation process: (1) cavitation within the amorphous layers, observed only in the neat HDPE at e ≈ 0.1–0.15, (2) microbuckling followed by development of lamellar kinks, e ≈ 0.3–0.4, and (3) slip instability leading to its localization and subsequent lamella fragmentation, e > 0.6. Cavitation is a phenomenon specific to tension, while two latter instabilities were also observed in other deformation modes.

The following conclusions can be drawn from this study:

- When cavitation is suppressed, plastic deformation in tension follows exactly the same path as it does in compression, using the same mechanisms and instabilities and resulting in very similar true stress–true strain relationships and structural evolution.
- Microbuckling and slip instability appeared to be common and important steps in deformation, occurring at similar strains in both compression and tension. They facilitate further deformation by opening new paths of deformation, more effective in strain accommodation than any previously available.
- It was confirmed that cavitation, frequently observed in tension, does not otherwise modify the deformation sequence to a significant extent. In particular, it does not prevent microbuckling instability, which was found in all materials.
- Slip localization instability can be triggered, in addition to lamella thickness nonuniformity, by stress inhomogeneities, e.g., stress concentrations generated at lamella faces by taut stress-transmitting chains or voids created in adjacent amorphous layers due to cavitation.
- The reduced concentration of ST chains in the HDPE/wax blend results in fewer but stronger stress concentrations than in chloroform-swollen HDPE, leading to increased slip localization and subsequent lamellae fragmentation.
- When cavitation occurs in the system, e.g., in neat HDPE, much stronger stress concentrations are generated at lamella faces, which greatly enhances lamella fragmentation, shifting it towards lower strains.
- Cavitation, creating nano- and micro-voids, accommodates a relatively small strain and does not change the lamella orientation. Hence, it cannot modify already active or activate new deformation mechanisms. Thus, it does not compete in a real way with microbuckling and slip instabilities, capable of opening new, more efficient deformation paths. Therefore, it can be considered a side effect, specific for tension, which, however, does not seem to be essential from the point of view of the overall deformation behavior.
- Cavitation should, however, be recognized as a serious and significant phenomenon, as it can notably increase lamella fragmentation and thus accelerate the transformation of the initial lamellar morphology into the final microfibrillar morphology. Cavitation also has a noticeable effect on the resulting properties and the appearance of the deformed material, introducing a significant number of voids into the structure.

## Figures and Tables

**Table 1:** The DSC and SAXS results obtained for non-deformed dry and swollen samples.

Sample	Concentration of Chloroform (vol.%)	T_m_ (°C) (DSC)	*X_c_* (wt.%) (*)	*X_v_* (vol.%) (*)	LP (nm) (SAXS)	*l_c_* (nm) (**)	*l_a_* (nm) (**)
HDPE—dry (starting material)	0	134.0	64.7	61.0	23.2	14.2	9.0
HDPE—swollen with chloroform	7.6	-	-	-	25.4		
HDPE—dried after swelling	0	133.8	64.6	60.9	23.0	14.0	9.0
HDPE/wax (6 wt.%)—dry	0	132.8	62.3	58.6	24.1	14.1	10
HDPE/wax (6 wt.%)—swollen with chloroform	7.3	-	-	-	25.4		
HDPE/wax—dried after swelling	0	133.5	62.8	59.1	24.0	14.2	9.8

(*): weight and volume crystallinity calculated from DSC data with Equations (4) and (5), respectively; (**): average crystalline and amorphous layer thickness *l_c_* and *l_a_* calculated from the long period LP determined from SAXS patterns and the DSC-based volume crystallinity: *l_c_* = LP *X_v_*/100%; *l_a_* = LP − *l_c_*.

**Table 2:** True strain at crossover points estimated from mechanical data.

Sample	True Strain at the Crossover Point:					
	1st		2nd	3rd		4th
	B: max. in σ_nom_	B’: Seen in S/S_o_	C: Seen in σ_true_	D: Seen in S/S_o_	D’: min. in σ_nom_	E: Seen in σ_true_
HDPE—neat (dry)	0.11	0.10	0.66	1.40	1.48	1.83
HDPE/chloroform	0.18	0.14	0.75	1.44	1.53	1.77
HDPE/wax (dry)	0.13	0.15	0.70	1.51	1.61	1.97
HDPE/wax–chloroform	0.19	0.15	0.69	1.49	1.68	1.91

## Data Availability

The original contributions presented in the study are included in the article, further inquiries can be directed to the corresponding author.
